# Investigation of the therapeutic effects and mechanisms of Houpo Mahuang Decoction on a mouse model of chronic obstructive pulmonary disease

**DOI:** 10.3389/fphar.2024.1448069

**Published:** 2024-11-07

**Authors:** Shanlan Li, Ziqi Dai, Tong Zhang, Zhuoqian Guo, Feng Gao, Xuehao Cheng, Jin An, Yixuan Lin, Xiaomin Xiong, Nan Wang, Guanghui Jiang, Bing Xu, Haimin Lei

**Affiliations:** ^1^ School of Chinese Pharmacy, Beijing University of Chinese Medicine, Beijing, China; ^2^ Aimin Pharmaceutical Group, Henan, China

**Keywords:** houpo mahuang decoction, chronic obstructive pulmonary disease, IL-17 signaling pathway, inflammation, transcriptomics, network pharmacology

## Abstract

**Background:**

With a growing global population affected by Chronic Obstructive Pulmonary Disease (COPD), the traditional Chinese herbal formula Houpo Mahuang Decoction (HPMHD) has been used for centuries to address respiratory ailments. While studies have demonstrated the therapeutic benefits of HPMHD in COPD, the effective active ingredients, potential targets, and molecular mechanisms underlying its effectiveness remained unclear.

**Methods:**

The mechanisms of action of certain HPMHD components, targets, and pathways for the treatment of COPD were predicted using a network pharmacology method. We induced a COPD mouse model using porcine pancreatic elastase and evaluated the pathological changes and healing processes through HE and Masson staining. Immunofluorescence was used to assess the levels of IL-6 and TNF-ɑ. RNA-Seq analysis was conducted to identify differentially expressed genes (DEGs) in the lungs of normal, control, and treated mice, revealing the biological pathways enriched by HPMHD in COPD treatment. Finally, the expression of DEGs was verified using Western blotting and RT-qPCR.

**Results:**

HPMHD effectively alleviated pathological symptoms and improved COPD in mice by modulating the IL-17 signaling pathway. Treatment with HPMHD improved lung morphology and structure, reduced inflammatory cell infiltration, and inhibited IL-6 and TNF-ɑ levels. Network pharmacology and transcriptomics further revealed the mechanism, indicating that the IL-17 signaling pathway might been instrumental in the inhibitory effect of HPMHD on mouse model of COPD. Subsequent experiments, including protein blotting and RT-qPCR analysis, confirmed the activation of the IL-17 signaling pathway by HPMHD in the COPD mouse model, further supporting the initial findings.

**Conclusion:**

HPMHD was shown to alleviate COPD and reduce lung inflammation in mice, potentially through the activation of the IL-17 signaling pathway. This study provides a novel direction for the development of COPD drugs.

## 1 Introduction

In 2020, chronic obstructive pulmonary disease (COPD), a serious respiratory illness, is expected to be the third leading cause of death (Caramori et al., 2014; Cross, 2005). Continuous airflow limitation, often worsening over time, is a key feature of the condition, stemming from persistent inflammation in the airways and lungs caused by exposure to harmful substances (Vestbo, 2014). Factors such as smoking and air pollution contribute to the development of COPD, making it a major global health concern affecting the wellbeing and mortality of individuals (Mannino, 2022). Dexamethasone (DXM), a synthetic glucocorticoid, has been used in clinical practice (Ren et al., 2016).

Traditional Chinese medicine (TCM) has gained recognition as an important therapeutic option for various chronic diseases worldwide, including COPD (Hu et al., 2022). Houpo Mahuang Decoction (HPMHD) is a classic traditional Chinese medicine prescription, documented in the Golden Chamber (Jin Gui Yao Lue) by Zhang Zhongjing in the Eastern Han Dynasty, has been used in China for over 2000 years. The treatment symptoms of HPMHD described in the Golden Chamber as “cough with a floating pulse,” and the Tang Dynasty Fangshu “Valuable Prescriptions for Emergency” expanded its treatment scope to “qi counterflow, chest tightness, wheeze”, which were corresponding to the indications described in the Global strategy for the diagnosis, management, and prevention of COPD (2020) (Singh et al., 2019). In recent years, HPMHD has been increasingly used in the clinic for the treatment of COPD, with no reported adverse effects (Li et al., 2021; Sun et al., 2022). However, the specific bioactive ingredients and molecular mechanisms underlying its therapeutic effects for COPD remained unclear.

In this research, we utilized network pharmacology to identify the active components of HPMHD and predict their target proteins. Additionally, we assessed the efficacy of HPMHD by examining the histopathologic changes in the lungs and the levels of IL-6 and TNF-α immunofluorescence. Furthermore, we employed high-throughput techniques and real-time quantitative reverse transcription polymerase chain reaction to identify differentially expressed miRNAs, and validated the associated proteins through Western blot. Our goal is to enhance understanding of HPMHD’s therapeutic potential for a mouse model of COPD and elucidate its underlying mechanisms.

## 2 Materials and animals

### 2.1 Materials and reagents

HPMHD decoction was provided by Aimin pharmaceutical group co., Ltd., and included the cortex of *Magnolia officinalis* Rehd. et Wils (69.0 g), the stem of *Ephedra sinica* Stapf (55.20 g), the rhizoma of *Asarum heterotropoides* Fr. Schmidt var. *Mandshuricum* (Maxim.)Kitag (27.6 g), the rhizoma of *Pinellia ternate* (Thunb.) Breit (34.5 g), *Gypsum Fibrosum* (90 g), the fructus of *Schisandra chinensis* (Turcz.) Baill (37.0 g), the semen of *Prunus armeniaca* L (57.0 g), the rhizoma of *Zingiber officinale* Rosc (27.6 g), and the fructus of *Triticum aestivum* L (140.0 g). Magnolol standard samples (lot: 110,729–202015, 99.0%), and Honokiol standard samples (lot: 110,730–201915, 99.8%), Ephedrine hydrochloride standard samples (lot: 171,241–201809, 100.0%), Pseudoephedrine hydrochloride standard samples (lot: 171,237–201510, 99.8%), Amygdalin standard samples (lot: 110,820–201808, 88.2%), Schisandrol A standard samples (lot: 110,857–201815, 99.7%), 6-gingerol standard samples (lot: 111,833–202007, 99.3%), and Asarinin standard samples (lot: 111,899–201705, 100.0%) were purchased from the National Institutes for Food and Drug Control. Chromatographically pure acetonitrile and methanol used in the HPLC method were purchased from Thermo Fisher Scientific (Thermo Fisher United States). The chromatographically pure phosphoric acid was purchased from Daimao Chemical Reagent Factory (Daimao Corp., China). The experimental water was purified water (Wahaha Corp., China). Porcine pancreatic elastase (PPE) was purchased from Sigma Aldrich (Shanghai) Trading Co., Ltd. Dexamethasone was purchased from Xi’an Kanghua Pharmaceutical Co., Ltd.

**FIGURE 1:**
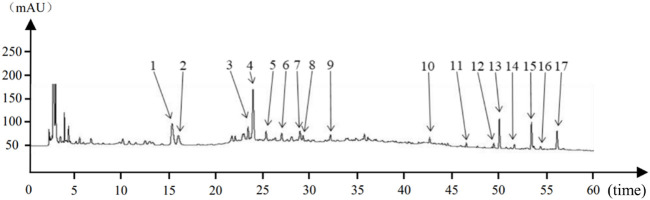
Houpo Mahuang Decoction Substance Reference Cross Reference Chart:1–17 indicated 17 characteristic peaks.

The preparation method of HPMHD extract was determined after our preliminary method investigation as follows: *T. aestivum* L. 8 times water boiling and keep slightly boiling decoction for 30 min. After that, the remaining herbs were soaked in *T. aestivum* L. liquid for 30 min, and after boiling, keep slightly boiling decoction for 120 min to the volume of decoction liquid was 600 mL. The decoction liquid was filtered and placed in −80°C pre-cooling for 12 h and then freeze-dried, which is to obtain the HPMHD lyophilized powder. This lyophilized powder was subsequently used in HPLC assays to identify characteristic components and treatments as detailed in Figure 1.

### 2.2 Apparatus and methods

#### 2.2.1 HPMHD substance baseline characterization mapping

##### 2.2.1.1 Preparation of HPMHD test solutions

HPMHD lyophilized powder (0.2 g) was dissolved in 75% methanol HPLC grade (25 mL) and then extracted by ultrasonication (250 W, 40 kHz) for 20 min, and then methanol reweighed and then filtered through a 0.22 μm microporous filter head, which was the test solution.

##### 2.2.1.2 Preparation of mixed standard solution

6.63 mg of ephedrine hydrochloride, 6.39 mg of pseudoephedrine hydrochloride, 6.81 mg of amygdalin and 2.30 mg of 6-gingerol were weighed precisely and put into a 10 mL vial, and 75% of methanol was added to the scale to obtain the control stock solution Ⅰ. Precisely weighed 10.16 mg of Schisandrin, 7.12 mg of Magnolol, 7.58 mg of Honokiol, and 7.62 mg of Asarinin in a 10 mL vial, added 75% methanol to the scale, and then sucked up 1 mL of it in a 10 mL vial, added 75% methanol to the scale to obtain the control stock solution Ⅱ. Precisely absorb 2 mL of control stock solution Ⅰ, Ⅱ 5 mL in a 50 mL measuring flask, add 75% methanol to the scale, 0.22 μm microporous filtration head filtration that is to obtain the mixed standard solution.

##### 2.2.1.3 HPLC analysis

The analysis was performed in an Ultimate 3,000 high performance liquid chromatograph (Thermo Fisher Scientific, United States). The separation was performed on a Kromasil 100-5-C18 column (250 mm × 4.6 mm, 5 μm). The mobile phase consisted of acetonitrile (A) and 0.15% aqueous phosphoric acid (B), and eluted according to the following gradient system (0–5 min, 5%–7% A; 5–15 min, 7%–8% A; 15–18 min, 8%–13% A; 18–29 min, 13%–20% A; 29–38 min, 20%–32% A; 38–46 min 32%–60% A; 46–55 min, 60%–72% A; 55–61 min, 72%–95% A; 61–71 min, 95%–95% A; 71–71.1 min, 95%–5% A; 71.1–86 min, 5%–5% A). The flow rate was 1.0 mL/min, the column temperature was 25°C, and the injection volume was 10 μL.

#### 2.2.2 Pharmacology in networks

Network pharmacology is a valuable tool in drug discovery that can provide a comprehensive understanding of how specific molecules interact with biological systems. By merging multiple omics methods, it allows for a functional-level analysis of biological responses (Boezio et al., 2017). In this study, we utilized network pharmacology to forecast how HPMHD and COPD might interact.

##### 2.2.2.1 Targets screening of compounds in HPMHD

OB represents the proportion of a drug taken orally that enters the systemic bloodstream. In drug screening, it is one of the most often utilized pharmacokinetic features. DL is a molecular parameter that measures how drug molecules are absorbed, distributed, metabolized, and excreted and is a reflection of their pharmacokinetics. In this study, those components with OB ≥ 30% and DL ≥ 0.18 were chosen as the active ingredients for the next step. These criteria ensured that the active ingredients had a higher likelihood of reaching their target sites and exerting their therapeutic effects. Subsequently, we used target fishing based on the candidate ingredients to identify the potential targets. We retrieved the small molecular structure data of the active ingredients in HPMHD from the PubChem database (https://pubchem.ncbi.nlm.nih.gov/) and employed the Swiss Target Prediction webserver (http://swisstargetprediction.ch/) for target screening.

##### 2.2.2.2 Targets retrieve of COPD

To find COPD targets, the term “chronic obstructive pulmonary disease” was retrieved from GeneCards (https://www.genecards.org/), TTD (https://db.idrblab.net/ttd/) and DrugBank database (https://go.drugbank.com/).

##### 2.2.2.3 Targets linked to COPD and identified compounds

To investigate the molecular mechanisms underlying the therapeutic effects of HPMHD for COPD, we utilized the Jvenn platform to generate a Venn diagram. This diagram depicted the intersections between the targets associated with COPD and the compounds identified in HPMHD. The resulting intersections were exported to Excel along with the names of all targets associated with the identified compounds and COPD. To construct a “compounds-targets disease” network, we employed the Cytoscape 3.9.1 software. And the common targets obtained from the Venn diagram were considered as seed nodes.

##### 2.2.2.4 PPI (protein-protein interaction) construction

Potential active ingredient targets of action for HPMHD and targets of action for COPD disease were imported into the VENN online tool and common targets were obtained. Then, we utilized the STRING database to establish a protein-protein interaction (PPI) network for these Jointly owned targets. The resulting PPI network was imported into Cytoscape 3.9.1 for visualization and analysis. The Network Analyzer plug-in was utilized to analyze the topological parameters of the network nodes. To identify the core targets of HPMHD for COPD treatment, we employed the screening criteria “degree > median, median centrality > median, centrality proximity > median”.

##### 2.2.2.5 Active compounds in HPMHD

We performed a topology analysis by converting the PPI network’s output to Cytoscape 3.9.1. The screening parameters were established by determining that degree should be at least 30, betweenness centrality should be at least the median, and closeness centrality should be at least the median. Nodes meeting all of these requirements were deemed suitable targets for HPMHD-assisted COPD treatment. By combining the compounds, effective targets, and disease, we constructed a “compounds-effective targets-disease” network.

##### 2.2.2.6 GO and KEGG pathway enrichment analyses

For GO enrichment analysis, we used DAVID (https://david.ncifcrf.gov/) (Sherman et al., 2022) and for pathway enrichment analysis, we used KEGG (https://www.kegg.jp/) (Kanehisa et al., 2023).

### 2.3 Animals

The animal study proposal was approved by the ethics committee of Beijing University of Chinese Medicine with the permit number 1121043000408. Following adaptive feeding, seven groups (n = 8) of sixty female BALB/c mice were randomly assigned: the Normal group, model group (COPD), HPMHD low-dose group (LHPHMD, 0.85 g. kg^-1^), medium-dose group (MHPMHD, 1.7 g. kg^-1^), high-dose group (HHPMHD, 3.4 g. kg^-1^) and dexamethasone tablet group (DXM) according to the random number table method. The mice in all groups, except the normal group, were sedated with pentobarbital sodium (60 mg. kg^-1^) and then intranasally administered with 30 μL of PBS solution containing 0.6 U of porcine pancreatic elastase (PPE) to induce COPD. From the 21st day of the experiment, the COPD and normal group were gavaged with 10 mL. kg^-1^ saline once daily. These treatment groups were orally administered low, medium, and high doses of Houpo Mahuang Decoction solutions (dissolved in saline), respectively. The positive control group received dexamethasone tablets at a daily dose of 0.002 g. kg^-1^, diluted in double saline and orally gavaged at a daily dose of 10 mL. kg^-1^ for 14 days.

### 2.4 Bronchopulmonary tissue sampling and processing

The thoracic and abdominal cavities were opened to fully expose the heart and lungs. Lung tissues were removed, and the left lung was fixed with 4% formaldehyde and embedded in paraffin, followed by Hematoxylin and Eosin staining (HE), Masson staining and immunofluorescence experiments. The right lung was kept at −80°C after being frozen with liquid nitrogen, followed by RT-qPCR and immunoblotting experiments for total protein.

### 2.5 RNA extraction and mRNA high-throughput sequencing

Total RNA was extracted from the lung tissue employing TRIzol®Reagent, adhering to the guidelines provided by the manufacturer. Purification of the isolated RNA was performed, followed by an evaluation of its purity and integrity using a Bioanalyzer 5,300 (Agilent, CA, United States), which had a concentration exceeding 30 ng μL^-1^ and an OD260/OD280 ratio of ≥1.8. The processes of RNA purification, reverse transcription, library preparation, and sequencing took place at Shanghai Majorbio Bio-pharm Biotechnology Co., Ltd (Shanghai, China) as per the guidelines provided by the producer (Illumina, San Diego, CA). After cDNA library construction, the cDNA libraries were quantified with Qubit 4.0, and then paired-end sequencing libraries were sequenced using a NovaSeq 6,000 sequencer (2 × 150 bp).

### 2.6 Comparative investigation of gene expression and functional enrichment

To determine genes that were differently expressed between the two samples, the transcript’s expression level was determined by using the transcripts per million reads (TPM) method. Gene abundances were quantified using RSEM (Li and Dewey, 2011). Differential expression analysis was executed with the use of either DESeq2 (Love et al., 2014) or DEGseq (Wang et al., 2010). DEGs with an absolute log2 fold change (|log2FC|) greater than or equal to 1 and a false discovery rate (FDR) less than or equal to 0.05 or 0.001 were considered significantly differentially expressed. Additionally, functional enrichment studies utilizing Kyoto Encyclopedia of Genes and Genomes (KEGG) and Gene Ontology (GO) were conducted to identify DEGs that showed significant enrichment in metabolic pathways and GO categories in relation to the transcriptome context as a whole. The significance level for the Bonferroni-corrected *p*-value was established at 0.05. Goatools and KOBAS (Xie et al., 2011) were used for GO functional enrichment and KEGG pathway analysis, respectively.

### 2.7 RT-qPCR for target-related DEGs

Complete RNA was isolated from pulmonary tissue via an RNA extraction kit (Majorbio Technology Co., Ltd., Shanghai, China) and transformed into cDNA using a FastKing RT kit (Majorbio Technology Co., Ltd., Shanghai, China). The primer sequencing for the target genes is listed in Table 1. The SYBR Green Promix Pro Taq HS qPCR kit (Accu-rate Biotechnology (Human) Co., Ltd., Changsha, China) was utilized for real-time quantitative PCR, in accordance with the manufacturer’s instructions. The CFX Connect Real-Time System (Bio-Rad, Hercules, CA, United States) was employed to track the fluorescence produced in the amplification process. GAPDH, the reference gene, served as the standard for normalization, whereas the 2^−ΔΔCT^ method was used to determine the target genes' relative expression levels.

**TABLE 1:** Real-time PCR primer sequence.

Gene	Primer sequence	
	Forward (5′–3′)	Reverse (5′–3′)
Cebpb	GCC​AAG​AAG​ACG​GTG​GAC​AAG	TGA​ACA​AGT​TCC​GCA​GGG​TG
il17rc	TCC​AGG​AGT​CTA​GGA​ACG​C	AAATACCGCAGAACCCAC
GAPDH	AGCCTCGTCCCGTAGACA	CTCGCTCCTGGAAGATGG

### 2.8 Western blotting

The lung tissues underwent lysis using RIPA buffer (CWBIO, China), followed by centrifugation and measurement with a BCA assay kit (CWBIO, China). Then 20 μg of protein samples were separated using 12% SDS-PAGE, transferred to PVDF membranes (Milli-pore, United States), blocked with protein-free rapid blocking buffer (Epizyme, China), incubated overnight with primary antibodies against IL-17 A (Proteintech Group, Inc., Wuhan, China, 66,148-1-lg), TNF-α (Proteintech Group, Inc., Wuhan, China, 26405-1-AP), and GAPDH (Proteintech Group, Inc., Wuhan, China, 60,004-1-lg), and incubated with HRP-coupled secondary antibodies (Proteintech Group, Inc., Wuhan, China, SA00001-1). Finally, Bio-Rad imaging system was utilized to visualize protein bands through the use of an ECL kit (New cell and Molecular Biotech Co., Ltd., China).

### 2.9 Statistical analysis

Data were represented as mean ± SD. Statistical significance was calculated by one-way ANOVA analysis among multiple groups followed by Tukey’s multiple comparison test. A *p*-value below 0.05 was deemed to indicate a statistically significant disparity. Every statistical evaluation was conducted using the GraphPad Prism 8.0.1 statistical program.

## 3 Result

### 3.1 Identification of active ingredients in HPMHD

**FIGURE 2:**
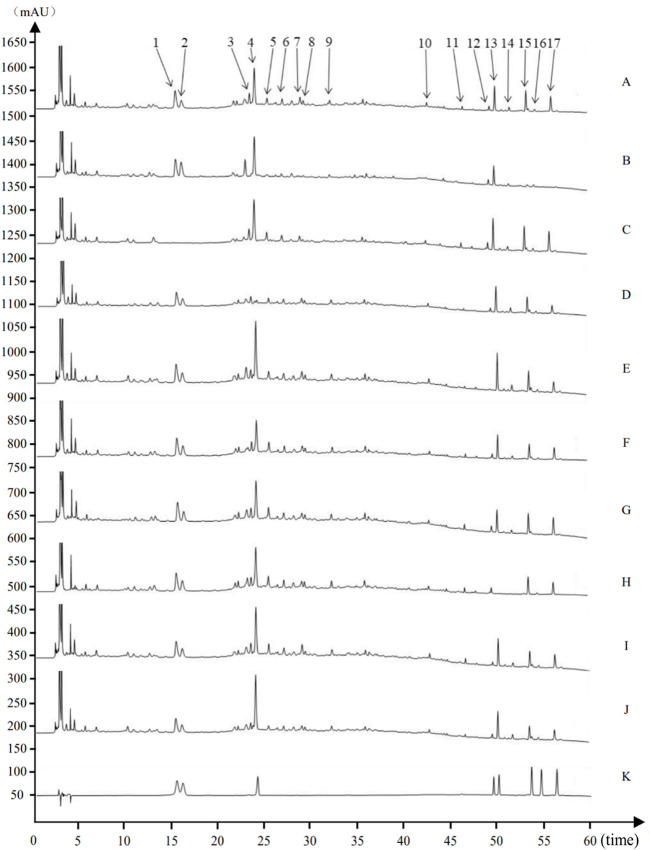
Source attribution of the characteristic peaks of the characteristic map of Houpo Mahuang Decoction **(A)** Test sample **(B)** *Magnolia officinalis* Rehd. et Wils. Negative control **(C)** *Ephedra sinica* Stapf negative control **(D)** *Prunus armeniaca* L. negative control **(E)** *Zingiber officinale* Rosc. Negative control **(F)** *Pinellia ternate* (Thunb.) Breit. Negative control **(G)** *Asarum heterotropoides* Fr. Schmidt var. *Mandshuricum* (Maxim.)Kitag. Negative control **(H)** *Schisandra chinensis* (Turcz.) Baill. Negative control **(I)** *Gypsum Fibrosum* negative control **(J)** *Triticum aestivum* L. negative control **(K)** Mixed Reference Standards. 1–17 indicated 17 characteristic peaks: (1) Ephedrine hydrochloride. (2) Pseudoephedrine hydrochloride. (4) Amygdalin (12) 6-gingerol (13) Schisandrin (15) Honokiol (16) Asarinin (17) Magnolol.

The eight characteristic components were characterized by HPLC. The mapping of HPMHD feature components and the botanical attributes of HPMHD feature components were shown in Figures 1, 2. The data indicated the presence of markers Ephedrine hydrochloride, Pseudoephedrine hydrochloride., Amygdalin, 6-gingerol, Schisandrol A, Honokiol, Asarinin, Magnolol.

### 3.2 Network pharmacology analysis of HPMHD

#### 3.2.1 Active ingredients of HPMHD and their targets against COPD

**FIGURE 3:**
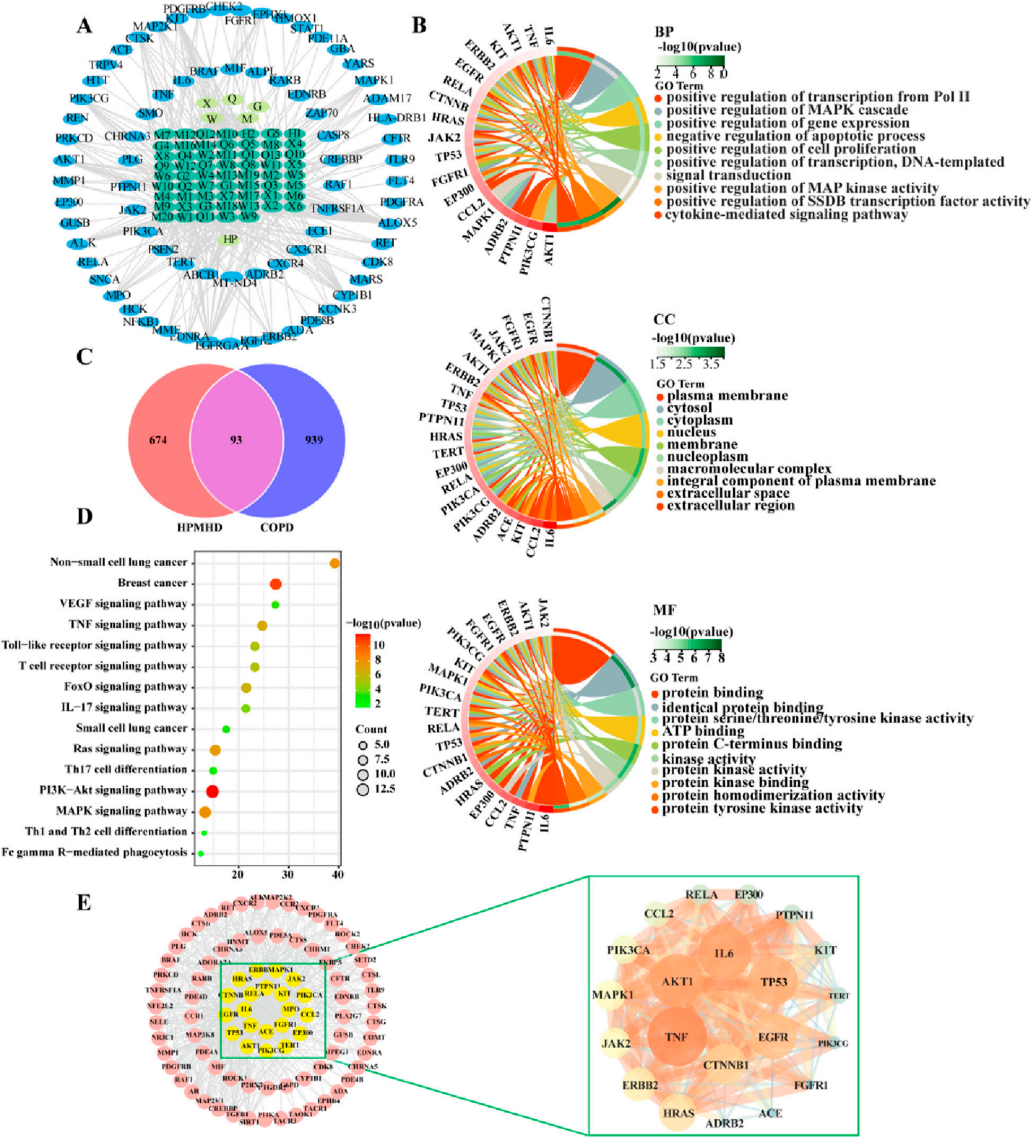
Network pharmacological predictions **(A)** “Herbs-ingredients-targets” network **(B)** GO enrichment analysis of targets of HPMHD compounds **(C)** Venn diagram **(D)** KEGG enrichment analysis of HPMHD compound action targets **(E)** Visualization analysis of core targets of HPMHD compound action.

Sixty-five active ingredients of HPMHD were extracted by TCMSP method. Additionally, 767 HPMHD active targets were obtained by high likelihood screening and repeated elimination based on these 65 active ingredients. To identify the targets related to COPD, we searched for relevant studies in the GeneCards database, the DrugBank database, and the TTD database, and obtained a total of 939 COPD targets. We then intersected the list of HPMHD targets with the list of COPD targets using a Venn diagram, resulting in a total of 93 therapeutic targets for HPMHD against COPD (Figure 3C).

#### 3.2.2 The network of “herbs-ingredients-targets,” KEGG pathway analysis, and GO enrichment

Cytoscape 3.9.1 was utilized to build a network map to visualize the relationships between herbs, components and targets (Figure 3A). The network consisted of 143 nodes and 417 edges, representing 75 genes and 61 active ingredients. These active components might play a critical role in the treatment of COPD through effective targets, which were ranked by their degree edges as shown in Table 2. We further analyzed these targets using KEGG pathway analysis, and the top 20 enriched signaling pathways were shown in Figure 3D. The results revealed that the main pathways targeted by HPMHD for the treatment of COPD included the non-small cell carcinoma cell signaling pathway, inflammation-associated TNF signaling pathway, and IL-17 signaling pathway. Additionally, we performed GO enrichment analysis and identified the top 20 enriched terms in biological process (BP), cellular component (CC), and molecular function (MF) categories (Figure 3B). The effective targets were found to be mainly involved in molecular functions such as membrane rafts, endomembrane systems, protein binding, and regulation of signaling receptors. They were also associated with biological processes such as ribonucleic acid transcription, regulatory activation of genes and protein kinases, and apoptosis of cell proliferation.

**TABLE 2:** Ranking of HPMHD active ingredient degree values.

Name	Degree	Name	Degree	Name	Degree
neokadsuranin	18	4, 9-dimethoxy-1-vinyl-β-carboline	10	10, 13-eicosadienoic	4
Kadsulignan C	17	Herbacetin	10	schizandronic acid	4
cyclo (L-tyrosyl-L-phenylalany)	16	1-Monolinolein	9	beta-D-Ribofuranoside, xanthine-9	4
Interiotherin B	15	neokadsuranic acid B	9	changnanic acid	3
Truflex OBP	14	campest-5-en-3beta-ol	9	24-Ethylcholest-4-en-3-one	3
(3S)-7-hydroxy-3-(2,3,4-trimethoxyphenyl)chroman-4-one	14	Cavidine	9	Baicalin	3
Pectolinarigenin	12	schisanlactone A	8	24-Ethylcholest-4-en-3-one	3
Cryptopin	12	kadsulignan B	8	sesamin	2
angusifolin B	12	Resivit	7	(−)-Asarinin	2
Caribine	12	Diosmetin	7	Neohesperidin	1
Sexangularetin	12	(+)-Leucocyanidin	7	Cycloartenol	1
Supraene	11	[(1S)-3-[(E)-but-2-enyl]-2-methyl-4-oxo-1-cyclopent-2-enyl] (1R,3R)-3-[(E)-3-methoxy-2-methyl-3-oxoprop-1-enyl]-2,2-dimethylcyclopropane-1-carboxylate	6	eriodictyol	1
luteolin	11	kadsulactone	6	beta-sitosterol	1
baicalein	10	neokadsuranic acid A	6	naringenin	1
delphinidin	10	neokadsuranic acid C	5	Stigmasterol	1
kaempferol	10	schisanlactone E	5	Eucalyptol	1
quercetin	10	coniferin	5	sitosterol	1
Genkwanin	10	12,13-epoxy-9-hydroxynonadeca-7,10-dienoic acid	4	poriferast-5-en-3beta-ol	1

#### 3.2.3 Construction of PPI networks and key Anti-COPD targets and components of HPMHD

The targets of HPMHD against COPD obtained from Venn diagrams were uploaded to the string database. And these targets with a confidence level greater than 0.95 were then selected for interaction. The resulting protein-protein interaction (PPI) network consisted of 93 targets, representing the main anti-COPD targets of HPMHD. To identify key targets, the obtained PPI network was evaluated using Degree, Closeness and Betweenness of Cytoscape 3.9.1 plug-in model Centiscape 2.2. This analysis revealed several key anti-COPD targets of HPMHD, including IL-6, TNF, MAPK1, EGFR, TP53, and CTNNB1 (Figure 3E).

### 3.3 Effect of HPMHD on body weight and pathological changes in mouse model of COPD

**FIGURE 4:**
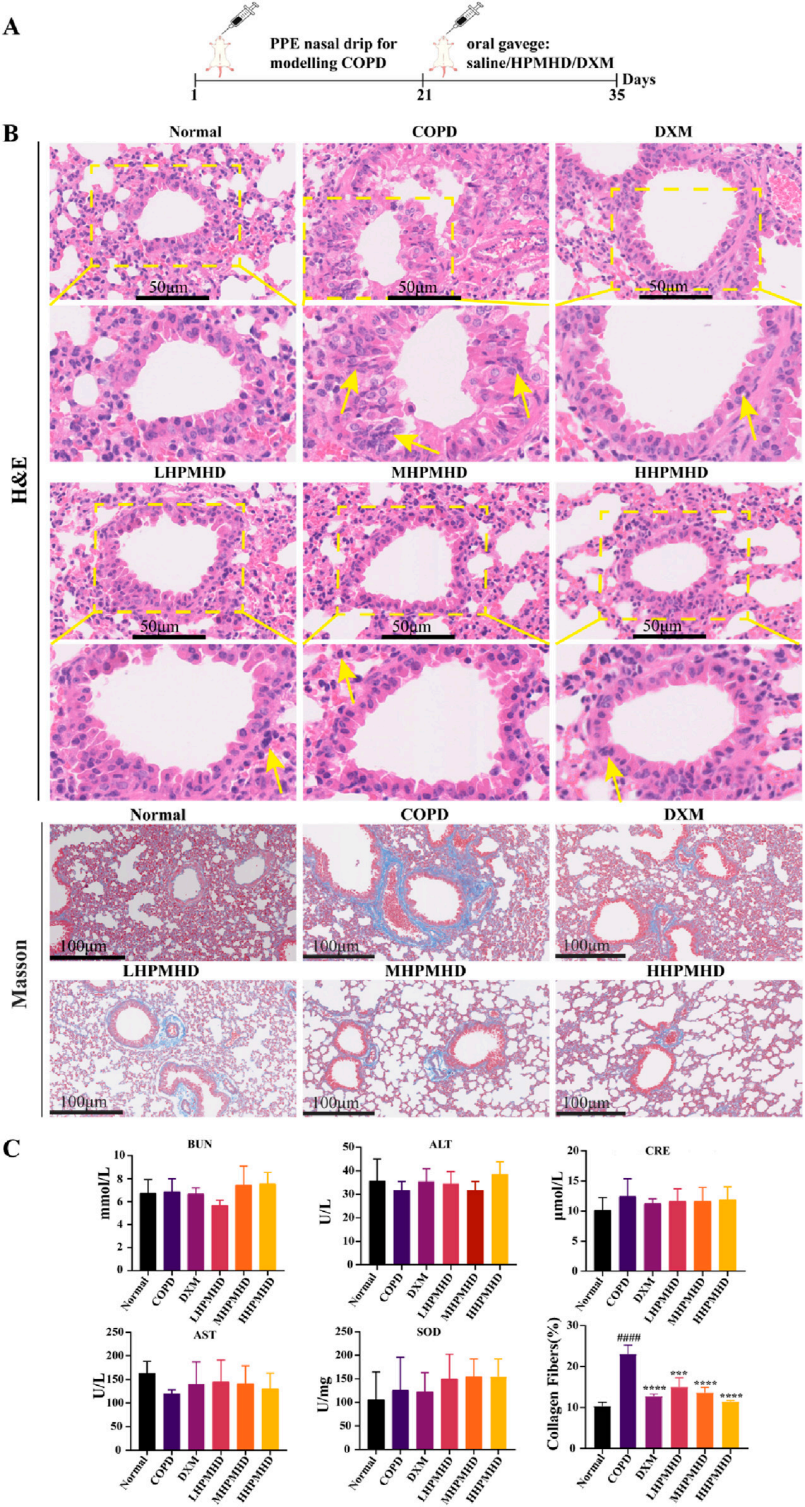
Histopathological changes in the lungs **(A)** Experimental design **(B)** Histopathological changes in the lungs (HE and Masson staining) **(C)** Toxicity of HPMHD to the liver and kidneys **(D)** Percentage of collagen fiber content in lung parenchyma, ^####^ *p* < 0.0001 *versus* normal mice; ^***^ *p* < 0.0005, ^****^ *p* < 0.0001 *versus* COPD group mice.

**FIGURE 5:**
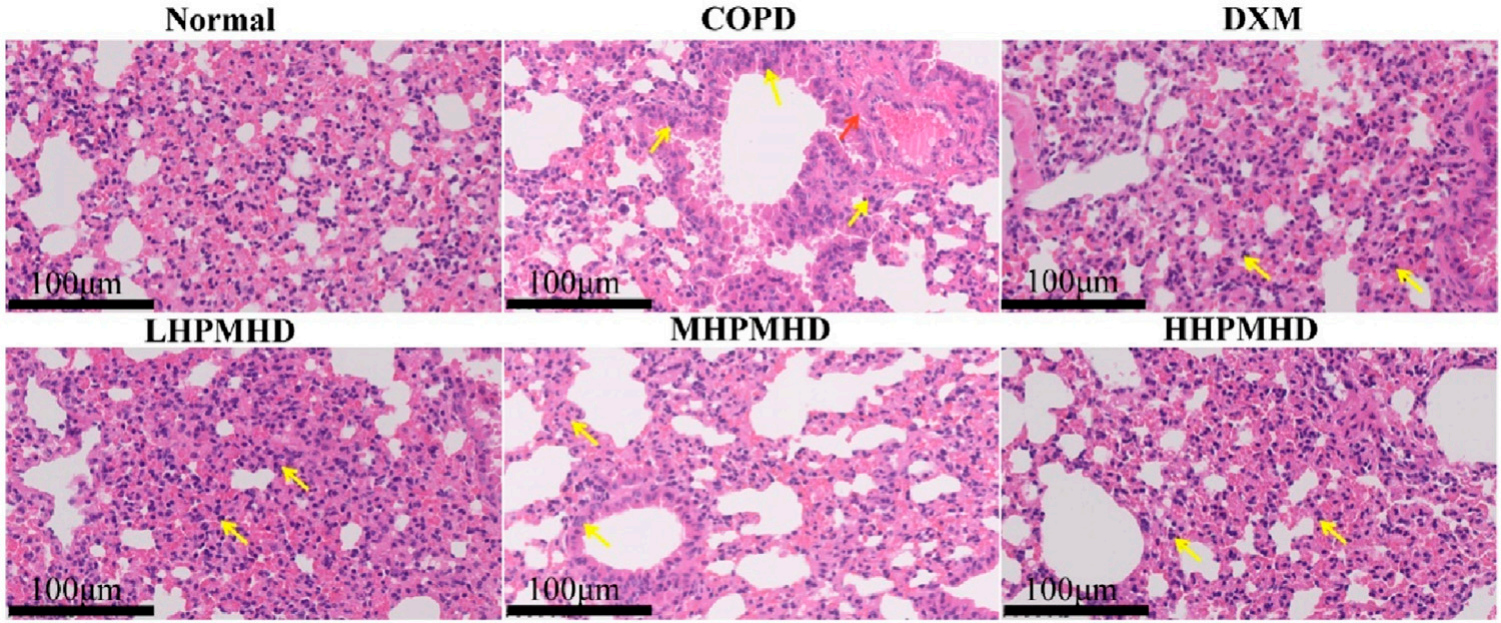
Histopathologic changes in the lungs (low magnification).

We performed HE and Masson staining of lung tissues to examine the pathological changes in a mouse model of COPD. The results indicated that HPMHD effectively improved COPD pathology. As shown in Figures 4B, 5, HE staining demonstrated that alveolar congestion with inflammatory cell (Yellow arrows) infiltration and increased mucus secretion was recognized in the COPD group compared with the normal group. In addition, the alveolar wall septa were significantly thickened. Treatment with DXM and HPMHD for 14 days significantly reduced alveolar congestion, inflammatory cell infiltration, mucus exudation, and restored alveolar wall integrity.

**FIGURE 6:**
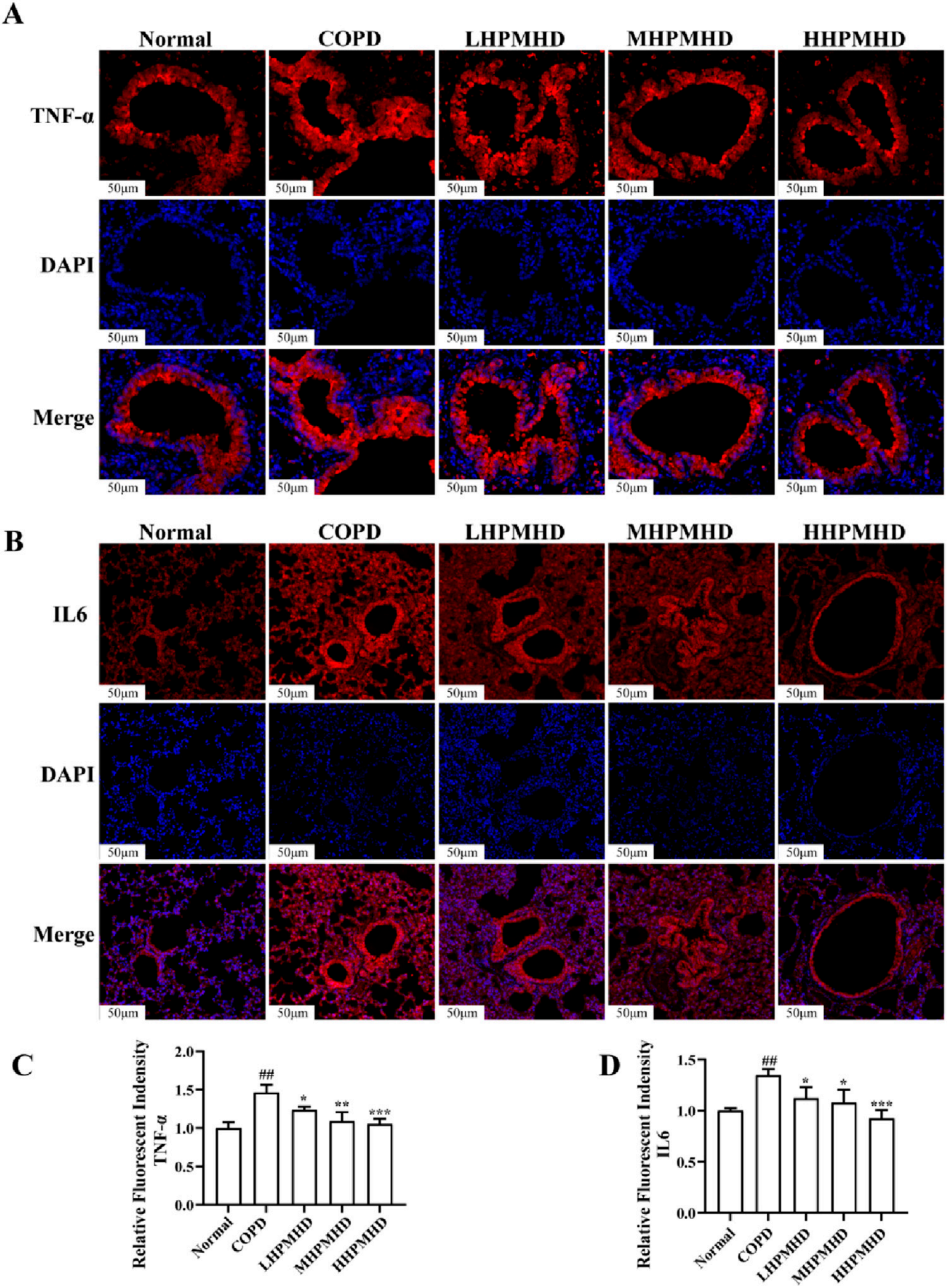
HPMHD reduced levels of IL-6 and TNF-α in lung tissue **(A)** Immunofluorescence images of TNF-α staining in the lungs **(B)** The statistical outcomes of **(A) (C)** Immunofluorescence images of IL-6 staining in the lung **(D)** The statistical outcomes of C, ^##^ *p* < 0.005, ^###^ *p* < 0.0005 *versus* normal mice; ^*^ *p* < 0.05, ^**^ *p* < 0.005, ^***^ *p* < 0.0005 *versus* COPD group mice.

**FIGURE 7:**
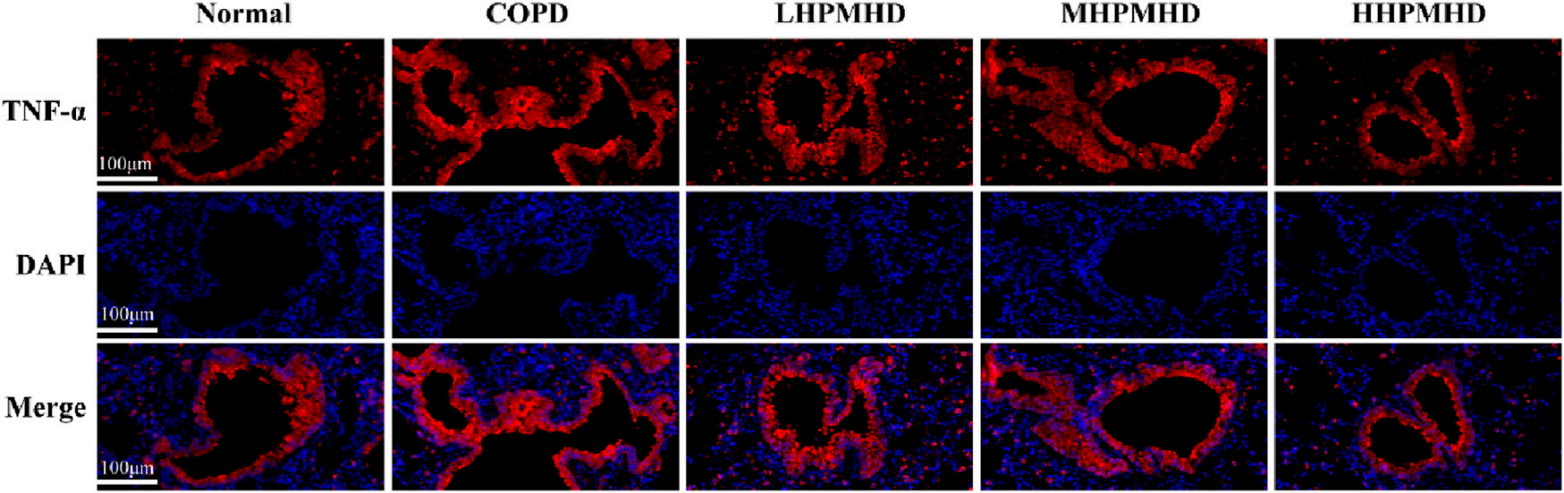
Immunofluorescence images of TNF-α staining of the lungs (low magnification).

Masson staining showed diffuse collagen fiber distribution in the COPD group, which was attenuated in the DXM, LHPMHD, MHPMHD, and HHPMHD groups (Figure 4B). The study of liver function and renal function showed that HPMHD showed high safety and no obvious toxicity (Figure 4C). Quantitative analysis confirmed HPMHD’s reduction in collagen deposition (Figure 4D). Immunofluorescence analysis of TNF-α and IL-6 expression in COPD mouse model revealed bright expression in the COPD group, with varying degrees of attenuation in each group following HPMHD treatment (Figures 6, 7).

### 3.4 Transcriptomics analysis

**FIGURE 8:**
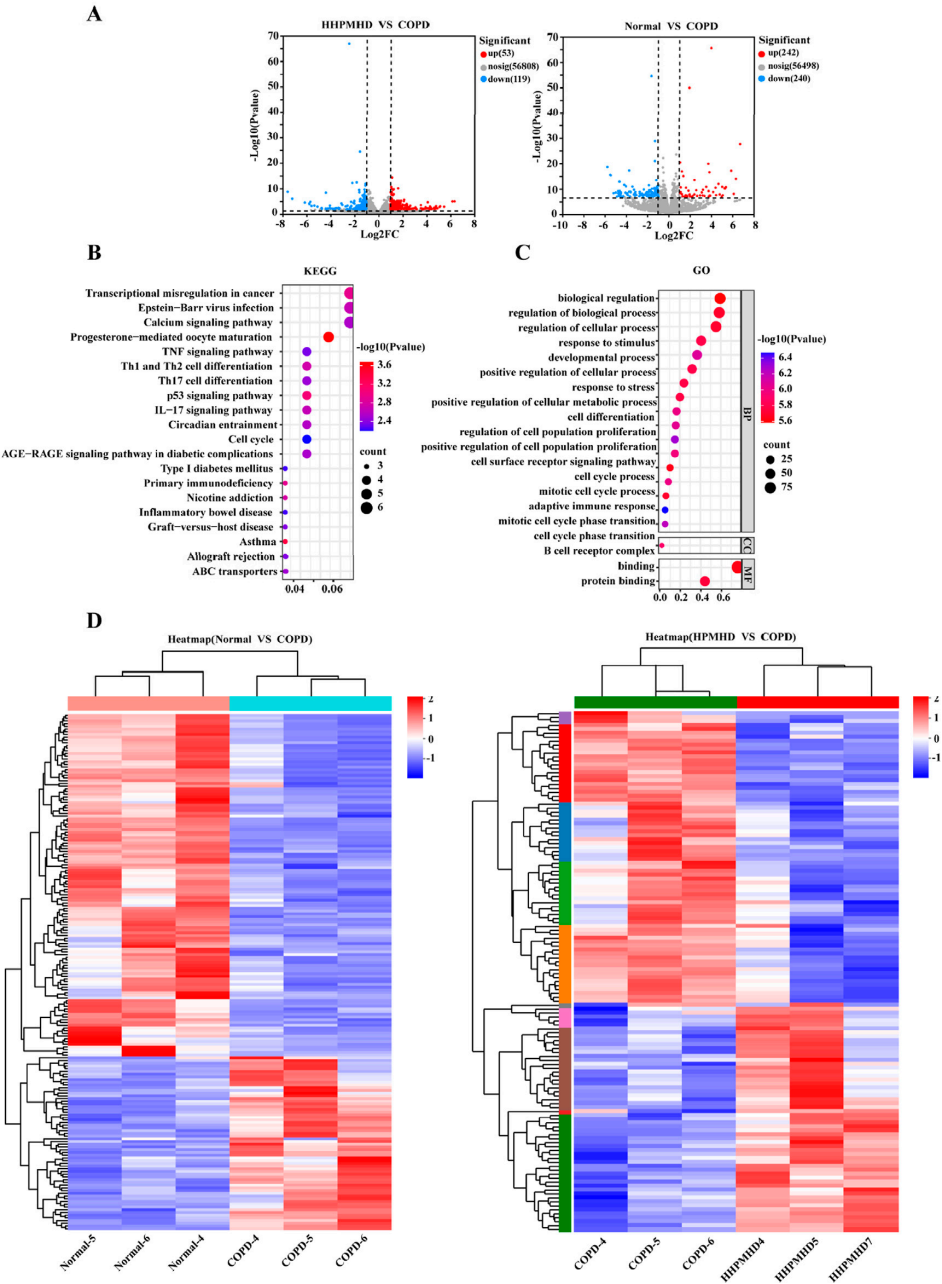
Transcriptomic analysis revealed signaling pathways involved in the inhibitory effect of HPMHD on mouse model of COPD **(A)** Volcano plots showing the number of DEGs in the normal group *versus* the COPD group and HPMHD **(B)** KEGG enrichment analysis of HPMHD therapeutic targets **(C)** GO enrichment analysis of HPMHD therapeutic targets **(D)** Heat map. Blue color indicates downregulated genes and red color indicates upregulated genes (n = 3).

To further illustrate the potential mechanisms of HPMHD on mouse model of COPD, we performed transcriptomic analysis using lung tissue. The volcano plot analysis revealed a total of 172 differentially expressed genes (DEGs) between the COPD and normal groups, with 53 upregulated and 119 downregulated genes. Additionally, we found 482 DEGs that changed more than 1-fold at a *p*-value ≤0.05 between the COPD and HPMHD-H groups, with 242 upregulated and 240 downregulated genes (Figure 8A).

To analyze the DEGs, we performed a heat map analysis (Figure 8D) and selected genes for further KEGG and GO enrichment analyses. The GO enrichment analysis indicated that these genes were involved in cellular and metabolic processes, with a focus on cell proliferation and differentiation, cell surface receptor signaling pathways, and inflammatory cytokines (Figure 8C). KEGG pathway analysis revealed that the therapeutic targets of HPMHD-H were mainly enriched in inflammation-related pathways, including the Th17 cell differentiation pathway, the IL-17 signaling pathway, the Th1 and Th2 cell differentiation pathway, and the TNF signaling pathway, among others (Figure 8B).

All these results collectively implied that the inhibitory effect of HPMHD-H on mouse model of COPD might be related to inflammatory metabolic pathways. These findings suggested that the inhibitory effect of HPMHD against mouse model of COPD might be mediated through inflammatory metabolic pathways. These results were aligned with the conclusions drawn from the network pharmacology analysis.

### 3.5 Network pharmacology and transcriptomics jointly reveal mechanisms of COPD inhibition by HPMHD

In order to obtain a deeper comprehension of the mechanisms behind HPMHD’s ability to improve mouse model of COPD, this study employed a combination of network pharmacology and transcriptomics analysis. The results revealed that the KEGG signaling pathway identified through network pharmacology was consistent with the pathway enriched by transcriptomics analysis. The key targets IL-6, TNF-α, and MAPK identified through network pharmacology were found to be enriched in the IL-17 pathway according to transcriptomics analysis. These findings suggested that the IL-17 signaling pathway might be accountable for the inhibitory effects of HPMHD on mouse model of COPD.

### 3.6 HPMHD inhibits IL-17 signaling pathway and expression of related genes and proteins

**FIGURE 9:**
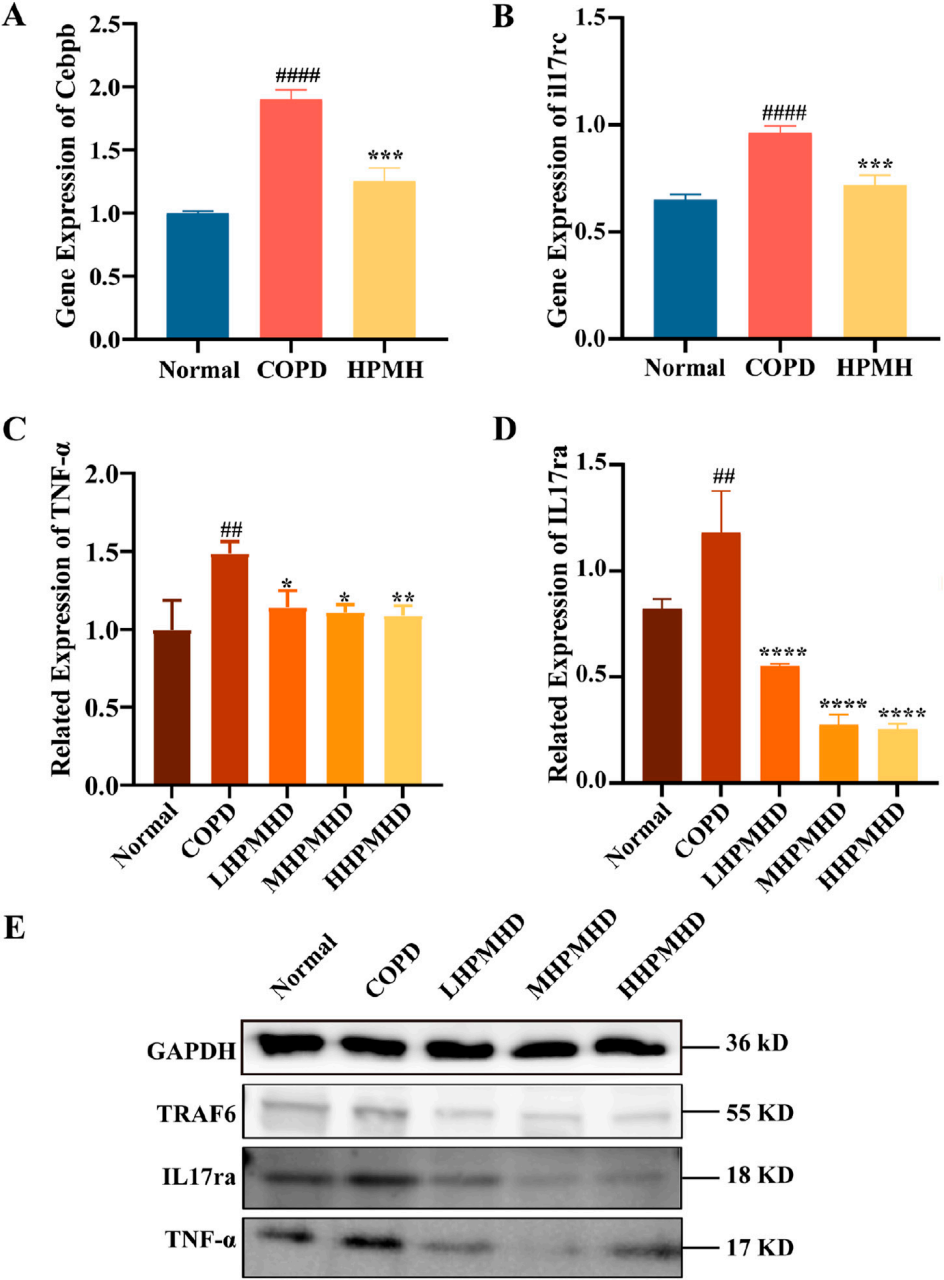
Validation of HPMHD inhibition of IL-17 signaling pathway in lung tissue **(A)** Expression levels of Cebpb analyzed by RT-qPCR **(B)** Expression levels of il17rc analyzed by RT-qPCR **(C)** Images of TNF-α protein levels determined by Western blotting **(D)** Images of IL-17ra protein levels determined by Western blotting **(E)** WB Result Images. Statistical analyses of the data were signified as the mean ± SD (n = 3), ^#^ *p* < 0.05, ^###^ *p* < 0.005 *versus* normal mice; ^*^ *p* < 0.05, ^**^ *p* < 0.005, ^***^ *p* < 0.0005, ^****^ *p* < 0.0001 *versus* COPD group mice.

The interleukin 17 (IL17) family plays crucial roles in acute and chronic inflammatory responses. IL-17 A is a key regulator of neutrophil inflammation in airway infections and is implicated in chronic lung diseases like asthma, COPD, and cystic fibrosis (McAleer and Kolls, 2014). The IL-17 family transmitted signals through their respective receptors and could activate downstream pathways, including NF-kappa B, MAPK, and C/EBP-β, to stimulate the production of chemokines, cytokines, and antimicrobial peptides. C/EBP-β transcription factors regulate cellular differentiation, cell cycle progression, and the expression of cytokines and chemokines associated with COPD and emphysema (Mori et al., 2015; Yang et al., 2021). It has been suggested that impaired C/EBP-β translation might lead to impaired lung tissue renewal processes and reduced protein renewal, and potentially contribute to inflammation and lung parenchymal destruction (Miglino et al., 2012). In this study, we concluded that the IL-17 signaling pathway and the inhibited expression of il17rc, C/EBP-β and TNF-α genes may be responsible for the reversal of COPD by HPMHD, and the possibility was confirmed by RT-qPCR and Western blot. The results demonstrated that PPE substantially upregulated the protein levels of IL-17A and TNF-α and promoted the abnormal activation of IL-17 signaling pathway (Figure 9). However, HPMHD treatment reversed the upregulation of these protein expressions in the model group.

## 4 Discussion

COPD is a condition linked to inflammation that is triggered by smoking and environmental factors, leading to airway blockage and lung inflammation (Vij et al., 2018). In COPD, the damage to airways involves several alterations, including the movement of squamous cells, heightened mucus production due to enlarged submucosal glands, a rise in epithelial secretory cells, enlarged airspaces resulting from alveolar damage (emphysema), fibrosis around the bronchioles, thickening of smooth muscle, and an increase in inflammatory cells (Larsson, 2008). The inflammatory response in COPD leads to disruption and remodeling of airway and alveolar walls, proliferation of cup cells, and increased mucosal glands (Wang et al., 2018).

Although the therapeutic effect of HPMHD on COPD has been clinically demonstrated, its mechanism is not clear. Since traditional Chinese medicine is characterized by multi-components, multi-targets, and multi-methods of action, this study firstly analyzed the targets and active components of the TCM compounds by network pharmacology, and then explored the efficacy of HPMHD against mouse model of COPD *in vivo*. Finally, its efficacy and mechanism of action were explored by immunofluorescence, WB, and RT-qPCR.

In this study, we obtained some potential active ingredients that may be useful for HPMHD in the treatment of chronic obstructive pulmonary disease (COPD) through the “herbs-ingredients-targets” approach. Some of these ingredients such as delphinidin, kaempferol, quercetin have been shown to have a protective effect against lung tissue damage. The impact of delphinidin on Sae cells exposed to cigarette smoke extract (CSE) has been researched. The results indicated that exposure to CSE led to a threefold increase in IL-8 levels in the cells. However, the addition of delphinidin (1 μM) significantly reduced IL-8 levels (Flores et al., 2012). Quercetin pretreatment (100 mg/kg, orally) was found to reduce airway cholinergic responses, lung inflammation, and epidermal accumulation as well as CD8^+^ T cells, CD11b^+^/CD11c^+^ macrophages, and neutrophils in rhinovirus-induced COPD phenotype mice. In addition, quercetin pretreatment reduced the expression of inflammatory factors such as CCL3, IFN-γ, TNF-α, and IL17 (Farazuddin et al., 2018). In acute lung injury induced in mice using lipopolysaccharide, kaempferol inhibited increased polyubiquitination of the K63 linkage on TNF receptor-associated factor 6 (TRAF6) and interleukin-1 receptor-associated kinase 1 (IRK1), elevated TRAF6 and IRAK1, and decreased levels of the inflammatory cytokines IL-6 and TNF-α and NF-κB (Qian et al., 2019). In addition, Pectolinarigenin was able to inhibit NSCLC cell proliferation, promote apoptosis, and inhibit metastasis through the PTEN/PI3K/AKT signaling pathway, suggesting that Pectolinarigenin was a potential drug for NSCLC treatment (Xu et al., 2018). Therefore, the potential active ingredients listed in Table 2 have very promising prospects for development as new drugs for COPD in the future.

It has been suggested that lung inflammation in COPD might be influenced by levels of IL17, which directly increased actin levels associated with the nuclear factor-kappa B (NF-кB) signaling pathway in airways and alveolar walls (Dos Santos et al., 2018). As a result, targeting the NF-кB and IL17 pathways as therapeutic targets for the therapy of lung illness may be appealing (Camargo et al., 2017). In this study, we found that HPMHD improves mouse model of COPD by regulating the inflammatory response of IL17 signaling pathway. In this pathway, IL17 signals via an IL17RC and IL17RA heterodimeric receptor complex, leading to the recruitment of ubiquitin ligase adapter proteins Act1 and TRAF6 (Gu et al., 2013). This in turn induced the phosphorylation of C/EBP-β, which regulates inflammation and host defense genes in the airway epithelium (Cassel and Nord, 2003; Roos et al., 2012). A negative correlation between lung function and airway expression of C/EBP-β in cigarette-induced COPD has been observed, suggesting a role for C/EBP-β in disease progression (Mori et al., 2015). N.M further revealed that proteolytic degradation of elastin by C/EBP-β leaded to expansion of the airspace and development of emphysema (Miglino et al., 2012). And the targets of IL17, C/EBP-β, TNF-α, IL-6 and other inflammatory factors and chemokines aggregated in this pathway were validated in immunofluorescence, RT-qPCR, Western blot and other experiments, and the levels of these indexes were significantly elevated in the disease group, while they were significantly downregulated in the drug-delivery group. All these evidences suggest that the pathogenesis of COPD is closely related to the IL-17 pathway, and HPMHD ameliorates mouse model of COPD by inhibiting the expression of the IL-17 pathway. As well as targets of inflammatory factors and chemokines such as IL17, C/EBP-β, TNF-ɑ, and IL-6. Western blot and RT-qPCR revealed that HPMHD inhibited the level of IL17 and led to a decrease in the level of downstream signals Cebpb and TN-ɑ, further confirmed that COPD could be treated by modulating the IL-17 signaling pathway.

In addition, IL-6 is a cytokine generated by inflammatory cells and also by primary lung epithelial cells in response to various different stimulants including allergens, respiratory viruses, and physical activity (Bohmwald et al., 2019; Kyler and Jones, 2023; Sharif et al., 2018; Takizawa, 2005). For an extended period, IL-6, together with TNF-ɑ and IL-1β, the other two traditional inflammatory cytokines have been acknowledged as the key indicator of inflammation. In addition to being a pro-inflammatory marker, IL-6 was also an active factor that has been successfully targeted in some inflammatory disorders, such as rheumatoid arthritis, and that contributed to the pathophysiology of these conditions (Kishimoto, 2010). There are clinical studies demonstrated that IL-6 levels elevated in sputum from patients with COPD, and a time decay determination of predicted FEV1 percentage identified negatively correlated with lung function (Donaldson et al., 2005; Eickmeier et al., 2010; Grubek-Jaworska et al., 2012). A longitudinal study of inflammatory markers in patients with COPD over a 3 year period also demonstrated that elevated serum levels of IL-6, but not TNF-ɑ or IL-8 levels, predicted increased mortality in COPD (Celli et al., 2012). The selective association of these clinical outcomes with IL-6 suggested the possibility of IL-6 being a parameter of increased inflammation, might also be a potential indicator of lung epithelial damage. Another study has shown that IL-6 deficiency leaded to decreased production of IL-13, which leaded to decreased mucus production by airway epithelial cells (Donaldson et al., 2005). Increased mucus is precisely one of the characteristics of COPD, suggesting that this could be a potential mechanism for IL-6-promoted COPD (Neveu et al., 2009). Our immunofluorescence results also demonstrated that IL-6 levels in the COPD were significantly higher than those in the normal group, and that the reduction in its levels also had a dose-dependent relationship for the HPMHD treatment, further confirming the relationship between IL-6 and COPD disease targets and fully elucidating the target of HPMHD for reversing mouse model of COPD. Obviously, IL-6 might be one of the therapeutic targets for HPMHD.

## 5 Conclusion

In conclusion, we explored the therapeutic role of HPMHD in mouse model of COPD and examined the regulatory role of HPMHD in mouse model of COPD at the genetic level. The current study showed that HPMHD improved mouse model of COPD and inhibited the expression of related genes (il17rc, cebpb) and proteins (IL-17A, IL-6, and TNF-α). The results of transcriptomics analysis as well as *in virto* and *in vivo* validation experiments demonstrate that the anti-COPD effect of HPMHD may be linked to the inhibition of IL-17 signaling pathway. This study clarified the mechanism and approach of HPMHD in improving mouse model of COPD, and provided a new idea for the research and development of drugs for COPD.

## Data Availability

The original contributions presented in the study are included in the article/Supplementary Material, further inquiries can be directed to the corresponding authors.
