# Supplementary material for: Investigation of the therapeutic effects and mechanisms of Houpo Mahuang Decoction on a mouse model of chronic obstructive pulmonary disease
Source: Front Pharmacol. 2024 Nov 7;15:1448069. doi: 10.3389/fphar.2024.1448069 (PMC11578825; doi:10.3389/fphar.2024.1448069)
Supplement: Supplementary file 1 [file DataSheet1.docx]

Fig. S1
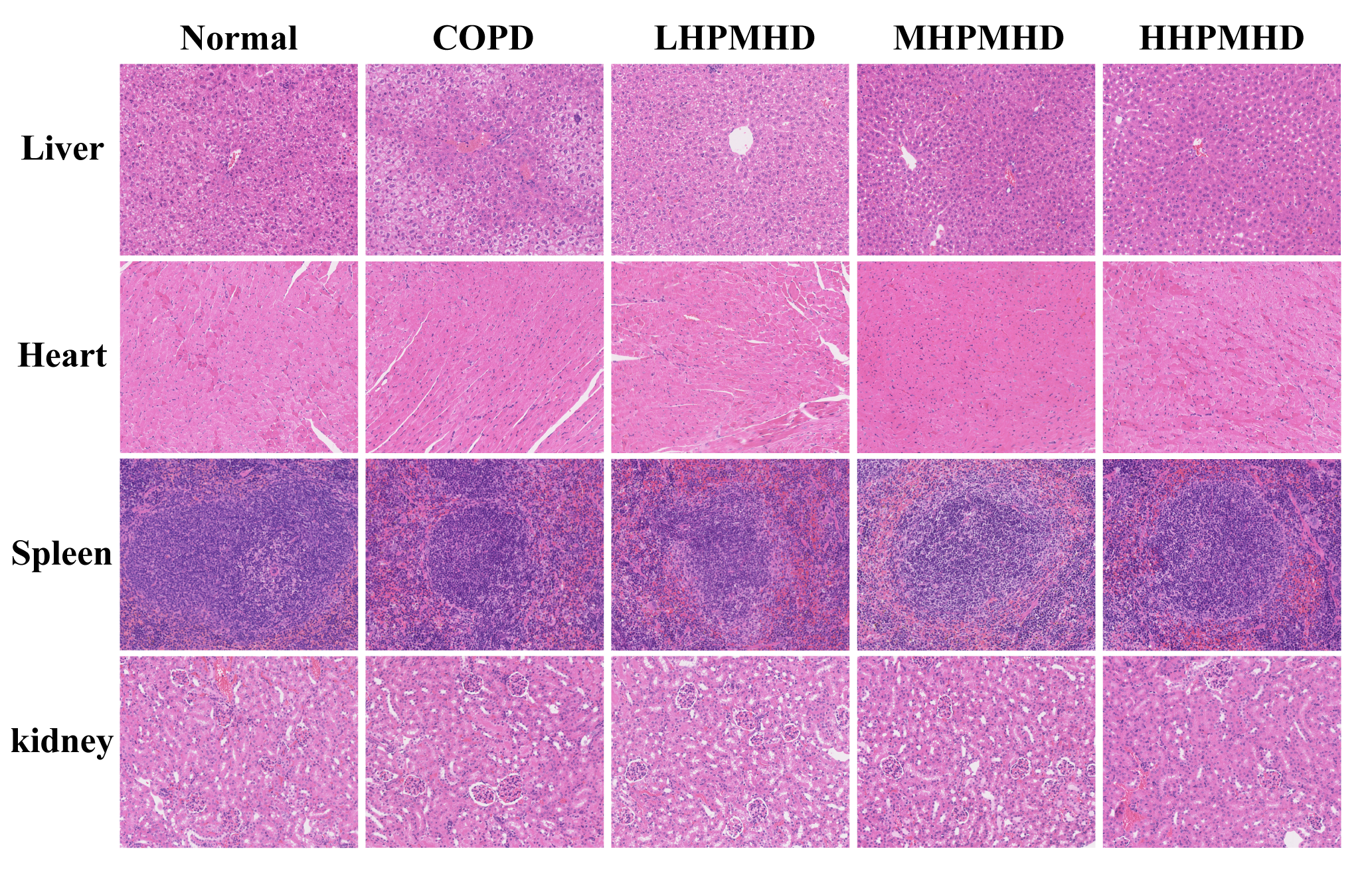
. HE sections of heart, liver, spleen and kidney.


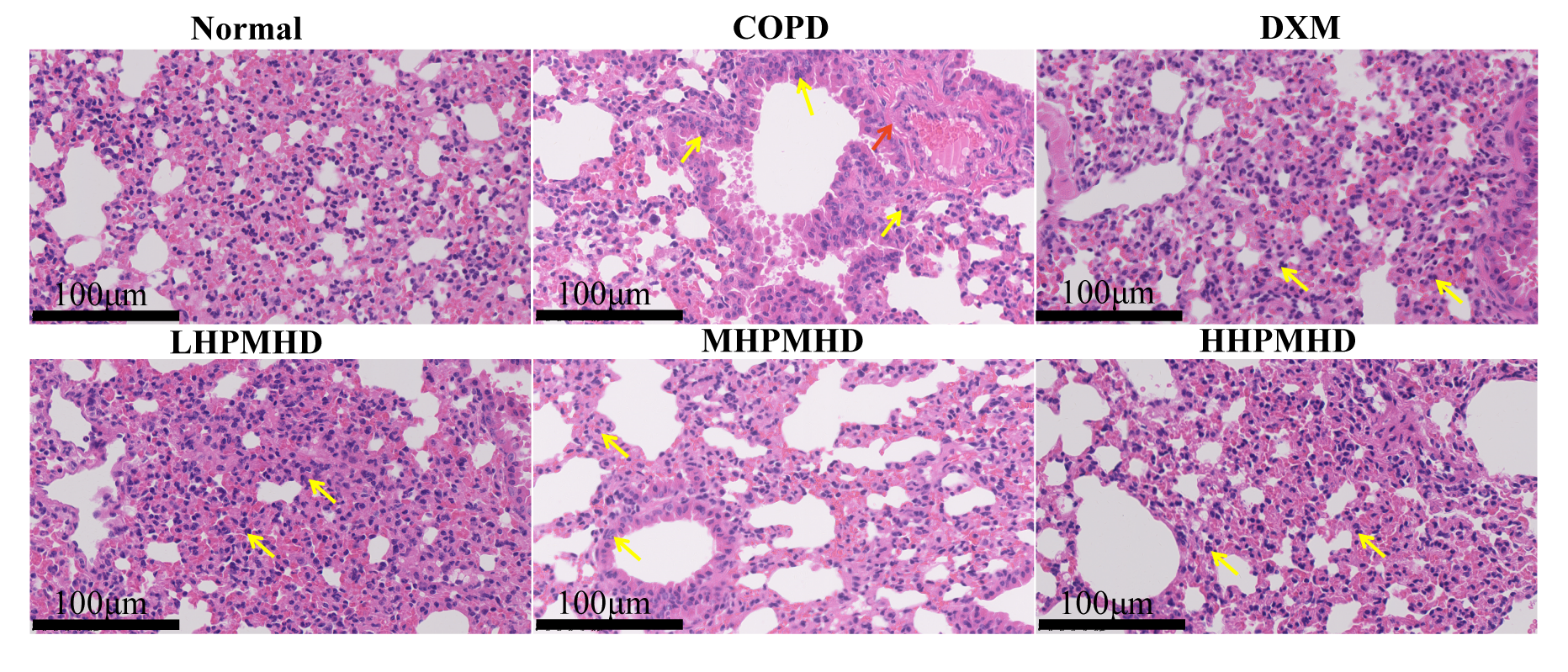


Fig. S2. Histopathological changes in the lungs.


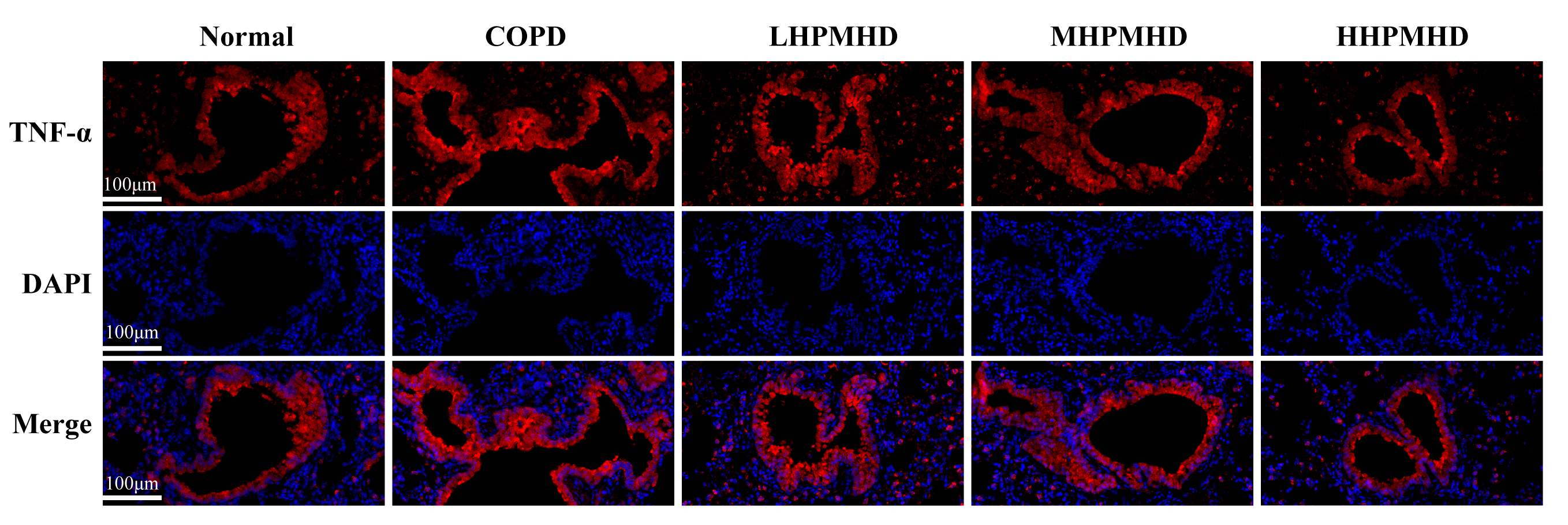


Fig. S3. Low-magnification images of TNF-α.
